# Power, fairness and trust: understanding and engaging with vaccine trial participants and communities in the setting up the EBOVAC-Salone vaccine trial in Sierra Leone

**DOI:** 10.1186/s12889-016-3799-x

**Published:** 2016-11-08

**Authors:** Luisa Enria, Shelley Lees, Elizabeth Smout, Thomas Mooney, Angus F. Tengbeh, Bailah Leigh, Brian Greenwood, Deborah Watson-Jones, Heidi Larson

**Affiliations:** 1 University of Bath, Claverton Down Road, Bath, BA2 7AY UK; 2 London School of Hygiene and Tropical Medicine, Keppel Street, London, WC1E 7HT UK; 3 College of Medicine and Allied Health Sciences (COMAHS), University of Sierra Leone, Fourah Bay College Campus, Freetown, Sierra Leone; 4 Mwanza Intervention Trials Unit, National Institute for Medical Research, Mwanza, Tanzania

**Keywords:** Vaccine trials, Ebola, Anthropology, Sierra Leone, Community engagement

## Abstract

**Background:**

This paper discusses the establishment of a clinical trial of an Ebola vaccine candidate in Kambia District, Northern Sierra Leone during the epidemic, and analyses the role of social science research in ensuring that lessons from the socio-political context, the recent experience of the Ebola outbreak, and learning from previous clinical trials were incorporated in the development of community engagement strategies. The paper aims to provide a case study of an integrated social science and communications system in the start-up phase of the clinical trial.

**Methods:**

The paper is based on qualitative research methods including ethnographic observation, interviews with trial participants and key stakeholder interviews.

**Results:**

Through the case study of EBOVAC Salone, the paper suggests ways in which research can be used to inform communication strategies before and during the setting up of the trial. It explores notions of power, fairness and trust emerging from analysis of the Sierra Leonean context and through ethnographic research, to reflect on three situations in which social scientists and community liaison officers worked together to ensure successful community engagement. Firstly, a section on “power” considers the pitfalls of considering communities as homogeneous and shows the importance of understanding intra-community power dynamics when engaging communities. Secondly, a section on “fairness” shows how local understandings of what is fair can help inform the design of volunteer recruitment strategies. Finally, a section on “trust” highlights how historically rooted rumours can be effectively addressed through active dialogue rather than through an approach focused on correcting misinformation.

**Conclusion:**

The paper firstly emphasises the value of social science in the setting up of clinical trials, in terms of providing an in depth understanding of context and social dynamics. Secondly, the paper suggests the importance of a close collaboration between research and community engagement to effectively confront political and social dynamics, especially in the context of an epidemic.

## Background

The Ebola epidemic in 2014 and 2015 in the West African countries of Guinea, Liberia and Sierra Leone was unprecedented in its size, over 28,000 people were infected and around 11,000 died [1]. In Sierra Leone, as of April 2016, 14,124 cases were registered, 3,956 of whom died [2]. As the epidemic took hold in Sierra Leone, Guinea and Liberia, reports were rife of instances of community resistance to medical intervention, communities’ mistrust and avoidance of healthcare centres, and stigmatisation of health workers and survivors [3–6].

Wilkinson and Leach argue that such resistance and mistrust must be understood through consideration of a long history of structural violence [7]. First as a central port in the Atlantic slave trade and then as a mining colony for the British Empire, a substantial proportion of Sierra Leone’s encounters with outsiders were primarily extractive [8, 9]. The country’s postcolonial past was similarly marred by years of oppressive and corrupt rule, which deprived the majority of the population of access to education, employment and basic healthcare services [10, 11] This was followed by a brutal ten year civil war (1991–2002), in which rebelling factions fought against authorities as symbols of a “rotten system”, but in the process all sides of the conflict systematically perpetrated violence against the civilian population [12–15]. Following the war and up until the Ebola epidemic erupted in 2014, the country was undergoing a long and difficult process of post-war reconstruction, during which citizens, government and foreign partners were attempting to build trustworthy governance structures at national and local level [10, 16]. The continuing poverty (Sierra Leone is ranked 181^st^ in the Human Development Index) and inequalities experienced by citizens and this deep rooted historical legacy of distrust in government and outside agencies can help explain challenges faced by those attempting to contain the spread of the Ebola virus [7].

The nature and rapidity of the spread of the disease also made clear the importance of understanding the socio-cultural dimensions of disease and the historical context which shapes them [7]. The initial response to the outbreak was undertaken by national government, national and international non-governmental organisations (NGOs), followed by the global UN led responses. Whilst prioritizing treatment centres, international organisations also focused their responses on surveillance, case management, safe burials, contact-tracing and community sensitization [17]. At first, interactions with communities focused on “sensitisation”, emphasising that local populations lacked knowledge on Ebola and that “traditional practices” spread disease [18]. As Chandler et al note, these strategies paid “little attention to the historical, political, economic, and social contexts in which they are delivered…[and] they reinforce external perceptions that local beliefs and practices are barriers to be overcome through persuasion or counterbalanced with incentives” ([18], p.1275).

As the epidemic developed and lessons were learned, examples emerged of changes in the engagement of local populations, as some aspects of the response adapted to deal more effectively with socio-cultural dimensions, taking seriously the importance of understanding communities’ needs and constraints and building trust. For example, safe burials that prevented people from washing dead bodies were made more acceptable by seeking the approval of local leaders, discussing the practices with the family of the deceased, burying the body in the presence of the community and including components of burial rituals such as Muslim shrouds on coffins [19]. The Ebola response’s learning curve was thus characterized by a gradual move away from attempts to correct misinformation towards the engagement of communities. In a recent analysis of the epidemic, Laverack and Manoncourt found that in a context of fear, mistrust and resistance, due to both the structural and political context as well as experiences of poor quality health care, utilising bottom up approaches to communication, which included a respect for local knowledge, was more effective [17]. As anthropologists working in these contexts have argued throughout the epidemic, rather than viewing customs and culture as barriers of resistance, it is more useful to consider how culture can adapt, something that, as Richards et al suggest, ought to be done in continuous consultation with local communities [20]. Reflecting on the West African Ebola epidemic, Abramowitz et al highlight how “under conditions of extreme stress, culture can be flexible and supple in response to extreme circumstances and the arrival of new information (such as public health messages), and make allowances in extraordinary conditions” ([21], p.4). The Ebola epidemic, in other words, rendered evident the benefits of engaging deeply with affected countries’ social, cultural and political context in order to understand communities’ response to the disease and to work with them to find ways to deal with crises.

The speed with which the epidemic grew and its extent demanded not only a rapid and coordinated response from international agencies and local authorities, but also called for the fast-tracked development of vaccines and other potential preventative and treatment technologies. This required the establishment of prophylactic vaccine trials in an epidemic context, which presents particular challenges for both the communities and the trialists. The setting up of such trials requires learning lessons from the historical and political context and from the experiences of the response to the outbreak, as described above, especially in terms of rooting communication strategies on deep understanding of socio-cultural contexts as adaptable and dynamic and on effective and active engagement of community members. In addition, the setting up of trials can benefit from learning from anthropological research on clinical trials related to other stigmatised diseases in resource poor contexts.

There is a growing body of work that is exploring complex political economy and social justice questions that emerge in communities where clinical trials are taking place [22–24]. These address broader ethical questions beyond the focus on informed consent. Molyneux and Geissler, for example, argue that this often does not take into account broader issues of inequities in wealth, health status and health care resources between the researchers and the researched ([24]; see also [22, 23, 25]). Dugas and Graham similarly suggest that standardised consent processes may be inadequate in contexts where translation is required, where socio-economic inequalities are stark and where collective consent may be important alongside individual consent [26]. Beyond these issues, there has been a growing body of research on the local effects of medical research on participants and their communities [24, 27–29]. Such research has revealed the salience of social relations developed between researchers and participants as well as the prevalence of rumours and concerns [28–36]. This body of research provides a foundation for developing strategies for engaging local populations and participants in a meaningful way grounded in the specificities of ethics and social relations in resource-poor contexts.

Given the nature of the Ebola epidemic, and the specific fears and concerns that the disease invokes, as well as the political history of Sierra Leone, the setting up of a vaccine trial during the Ebola epidemic required having an in-depth understanding of the epidemic and its effects as well as building trust within communities. With the prospect of such new encounters with biomedicine for people in communities with very little (and often no) experience of medical research, there is also an urgent need to determine what rumours, concerns, fears or mistrust emerge from dialogues with community members and individuals involved in vaccine trials and the ways in which such actors can be engaged in addressing these and building trust.

This paper discusses the role of social science (anthropology) and community liaison in supporting the establishment of the EBOVAC-Salone prophylactic Ebola vaccine trial in Kambia District, Northern Sierra Leone, during July to August 2015, towards the end of the Ebola epidemic. Through an exploration of notions of power, fairness and trust emerging from analysis of the Sierra Leonean context and through ethnographic research, the paper discusses three situations in which the EBOVAC-Salone social science and community liaison teams worked together to ensure effective communication, to develop appropriate recruitment strategies and to address rumours about the trial.

## The EBOVAC-Salone trial

In December 2014, the Innovative Medicines Initiative (IMI) awarded funding from the Ebola + programme to a consortia of research institutions to support the development, manufacturing and deployment of a prime-boost prophylactic Ebola vaccine regimen, in partnership with private industry [37]. A portion of the IMI2 funding awarded to the EBOVAC1 consortium is being used to implement the EBOVAC-Salone study in Sierra Leone. In addition to EBOVAC-Salone, IMI2 has also funded the related EBODAC (Ebola Vaccine Deployment, Acceptance and Compliance) consortium whose objective is to conduct community engagement and communication to support the prime-boost Ebola vaccine trials and roll-out of vaccines, if proven effective.

The EBOVAC-Salone study aims to assess the safety and immunogenicity of the Ad26.ZEBOV/MVA-BN-Filo prime-boost regimen in a population affected by Ebola. The study is being carried out in Kambia District in Northern Sierra Leone. Stage 1, an open label, safety and immunogenicity study of the prime boost vaccine in 43 healthy adults aged 18 years or older has completed the vaccination phase. The first participants to volunteer for the study were screened for eligibility on 20 September 2015 and vaccination began on 8 October 2015. In stage 2, a randomised, controlled, safety and immunogenicity study, 688 individuals will be screened and will be randomised to the prime boost vaccine or a control vaccine.^1^ Adults, adolescents and children aged one year or older will be included in this trial. Plans for additional stages are being finalized in consultation with the Sierra Leonean authorities and international health agencies. EBOVAC-Salone is coordinated by the London School of Hygiene &Tropical Medicine (LSHTM) working in partnership with Sierra Leone’s Ministry of Health and Sanitation and the College of Medicine and Allied Health Sciences. The EBOVAC-Salone study is one of several vaccine trials set up in West Africa during and in the immediate aftermath of the epidemic [38, 39].

## Methods: the community liaison and social science system

Engagement with the community in Kambia in the run-up to stage 1 of the EBOVAC-Salone study was conducted by a community liaison team and a social science team. The two teams were recruited locally.

The community liaison team, comprising nine locally-recruited staff, supervised by two LSHTM staff members, was responsible for implementing EBOVAC-Salone’s community engagement strategy. The team was complemented by an external communications manager who monitors rumours and concerns about the trial beyond the Kambia District and also provides information about the trial at national and international levels.

EBOVAC Salone was the first vaccine trial of any kind to have taken place in the study area, and the country as a whole has had limited experience of medical research. The community liaison team members thus received background training on clinical trials, with a particular emphasis on the difference between communication to support clinical trials and communication and social mobilisation for routine or proven interventions. The community engagement strategy for Stage 1 involved engaging with all levels of the community – from elected and traditional leaders to individual households - through a variety of channels. These included undertaking one-to-one engagement with key stakeholders, holding public meetings in partnership with influential civil society leaders, organising community meetings supported by local and traditional leaders, conducting house-to-house sensitisation visits, and hosting radio chat shows and phone-ins on local radio within Kambia District.

A Participant Advisory Group (PAG) was also set up, serving as a means through which study participants were encouraged to openly discuss their experiences of participating in the EBOVAC-Salone study with each other. The purpose of the latter group was to address concerns, share views, expose perceived barriers to enrolment and to share information where necessary. This group is entirely participant-led and at the first meeting the participants elected a Chairman and representative committee. For Stage 1, trial participants were invited to join the group by field workers who visited their homes in the initial seven days following vaccination. The frequency, structure and agendas of these meetings were decided by the group members themselves. The PAG is continuing to support stage 2, where 99 participants have enrolled to date.

The social science team was made up of four locally recruited research assistants, a data analyst and a transcriber, led by an LSHTM social scientist based in Kambia. The team was responsible for conducting anthropological research to examine community and participant perceptions and experiences of the EBOVAC Salone vaccine study, including any rumours and concerns around the trial and the vaccine. In addition, the team explored the socio-cultural context and perceptions of illness and disease in the community where Stage 1 was taking place. The methods used for this research were: ethnographic observation, focus group discussions, in depth interviews, and exit interviews with participants as they completed their visit to the vaccination clinic. Ethical approval for this study was granted by the ethics committees of LSHTM and Sierra Leone.

Ethnographic observations were carried out in key areas in Kambia and trial clinics to explore community dynamics; social interactions around the clinic; community narratives about EVD and the clinical trials; and to trace any ongoing rumours and concerns emerging at community level. Focus group discussions were carried out with both community members and with trial participants to discuss perceptions and experiences of the trial. In depth interviews were carried out with 6 Stage 1 Participants and 16 Stage 2 participants, interviewed after prime and boost vaccination, and with approximately 20 key stakeholders in Kambia. At the time of writing the Stage 2 research was ongoing. The data for Stage 1 was analysed through thematic and framework analysis. Framework analysis was used to answer the specific research objectives. A coding tree was developed according to the key research questions. Interview transcripts were coded according to this coding tree. Thematic analysis was used to develop emerging themes not identified by the research objectives.

The insights for this paper are based primarily on the ethnographic research carried out in Kambia in the three months preceding the opening of the trial. As the epidemic continued to ravage the country, and as Kambians lived under military curfew and constant news of Ebola cases, the trial team was renovating clinics, building laboratories and holding high-level conversations with key stakeholders. During the preparation phase for Stage 1, ethnographic encounters in local markets, *attaya* 2 bases, *poyo* 3 bars, shops and *okada* 4 parking grounds enabled the research team to develop a rich and complex picture of Kambia’s community dynamics, the everyday struggles and concerns of its inhabitants during the outbreak, their beliefs and fears about medical intervention and their views on the study that was about to open in their town.

## Results: communications and social science in action

During the preparation phase, the social science and community liaison teams developed a feedback system to inform rapidly research-driven communication strategies. Weekly meetings had three main purposes. Firstly, the social science team provided feedback on community engagement plans based on their research on the socio-cultural context, local community dynamics and perceptions of the vaccine trial. Secondly, the meetings brought up any issues encountered by the trial team or the community liaison staff that required further research by the social science team, such as the design of the recruitment strategies outlined below. Thirdly, the social science team reported on any rumours or concerns identified in the community. These rumours and concerns were communicated to the community liaison team anonymously in order not to breach confidentiality and to maintain the independence of the social science research. Following feedback, the community liaison staff brainstormed strategies to respond to concerns or rumours. Such strategies depended on the specific issue raised, but usually involved considering different and creative avenues for discussion with the community on the issue, reviewing messaging to actively engage with the issue at hand, and determining who the best person in the team was to respond and through which channel. When an urgent or potentially harmful rumour had been identified, this was reported immediately to the trial manager in Kambia and to the principal investigators.

This section of the paper discusses three examples of challenges faced by the trial team during the set-up phase of the study. These case studies reflect how lessons gained from knowledge of the country’s context, the experiences of the Ebola response and knowledge gained during previous trials helped inform the community engagement strategies and responses to issues as they came up. The examples are arranged through three key themes: power, fairness and trust. Each case study begins with a reflection on the context facing the trial team, then offers insights from the ethnographic research and finally considers the implications for community engagement strategies. These case studies elucidate the importance of research-driven mobilisation strategies, as well as the inevitable limitations on trialists’ ability to engage with all aspects of complex community dynamics.

### Power

The notion of “community” can often hide its heterogeneous nature and constant, underlying negotiations and contestations of power. The commonly used notion of ‘community’ used for example in public health campaigns and mobilization strategies can in fact hide complexities engendered by struggles over status, authority and economic resources. Questioning whose voice is heard, and the ways in which community ‘leadership’ is contested and in many ways produced by external interventions are at the heart of an understanding of communities as heterogeneous in and in flux. In Sierra Leone, Mariane Ferme has identified an accepted division between public and secret spheres, that is, a widely shared assumption that politics happens in different places, some public and some secret, and that covert strategies play a fundamental role in the working of politics [40]. This means that political intentions are permanently ambiguous, something that Ferme sees as “one of the defining features of postcolonial subjectivity in Sierra Leone” ([40], p.161). In what she defines as the “dialogics of publicity and secrecy”, overt acts of obedience are often paralleled by covert defiance. This first case study shows how these complicated, contextual and often hidden configurations of power and struggles over economic resources can impact on a vaccine trial’s mobilization strategy.

The challenges posed by community power dynamics became clear from the start of the engagement process. Community engagement for the EBOVAC Salone study began two months before the start of the trial with a series of community meetings, often chaired by various local authorities. The meetings were attended by the community liaison team, who presented information about the trial and who were ready to answer questions from the audience, and by the social science team, whose role was to observe, take notes and provide support if required. The meetings brought together groups of people identified as “key stakeholders”, as representatives of various important societal groups, such as area chiefs, market traders, teachers and civil society activists. At the meetings, stakeholders were provided with information about the trial and were given time to ask questions, raise concerns and offer suggestions.

A few days after one of the community meetings about the EBOVAC Salone Trial, the social science team was approached during daily ethnographic observations by a small group of ‘stakeholders’ who had attended one of the meetings. In exchange for the promise of absolute confidentiality, they asked that their concerns be heard. They reported that, while they had been asked in the EBOVAC Salone public meetings to discuss the vaccine trial with their communities and constituencies through smaller local meetings they felt unable to do so. They asked to meet at a separate time to explain their concerns. The next day the team met the group at one of their homes, and they began telling their story. They recounted how they felt that their position as respected leaders in their community had been undermined and that, as a consequence, their ability to mobilise their communities to participate in the trial was curtailed. They attributed the erosion of their power to the ‘selfishness’ of those they identified as ‘big’ leaders in Kambia, whom they blamed for failing to share the financial resources coming into the district. They voiced an assumption that powerful leaders must have ‘eaten’ significant amounts of Ebola funds before it could reach their communities.^5^ Emphasising the correlation between financial power and ability to command respect in their communities, these ‘small’ leaders argued that due to their economic struggles, they found themselves having to resort to borrowing money from their community members, which diminished their ability to command respect.

Regardless of the veracity or legitimacy of these allegations, these assumptions about the role of powerful authorities were widely reported during ethnographic research and they created significant amounts of discontent and quiet mistrust in authority figures in Kambia, as evidenced by the ‘small leaders’ private confessions. Despite dancing and clapping at public meetings, these ‘small’ leaders argued that they were unable and unwilling to act as spokespeople for local matters such as the vaccine trial, because they felt that their communities no longer respected them.

Although it was unclear whether this was in fact the reason for their decision to refrain from supporting the vaccine trial through the organisation of area meetings, the community leaders utilised these conversations to voice their dissatisfaction with the present structure of authority. Their arguments point to the importance of taking into account what are essential, but often hidden and unspoken, contestations of power within communities. The “dialogics between publicity and secrecy” exemplified by the reluctance to hold public meetings despite their public assertions of support, offers a salient example of the broader importance of taking into account the heterogeneity of community. Whilst community engagement meetings appeared to be received positively in Kambia, such manifest support could not be assumed to translate into community-wide acceptance. Insights such as those from the “small” leaders reveal that established power structures are not indisputably representative, nor are appointed leaders necessarily trusted by all sections of the population. Internal struggles over status and economic resources, as well as mistrust and disputes over rightful loci of authority must be taken into account. This cognizance of the contested nature of power undoubtedly creates its own challenges. Undoubtedly, having key and recognised stakeholders at the EBOVAC Salone community meetings was extremely valuable and its significance for a large number of Kambians cannot be underestimated. However, recognising that power is not always straightforward and that communities are fragmented is an important foundation for building more nuanced, sensitive and genuine engagement.

Ethnographic observation’s illustration of the deep-rooted contestations of power put forward by the “small” leaders and the political economy underpinning them, cannot be directly addressed by a community engagement team in a clinical trial. However, the social science and community liaison teams’ ability to identify such dynamics made it possible to initiate internal discussions about how to reconcile the importance of involving established authorities while diversifying engagement strategies to reach different sections of the community. This meant for example holding targeted sensitisation sessions in areas such as markets and popular meeting places, and holding radio talk shows in addition to key community leader-led meetings.

### Fairness

Notions of fairness are inevitably shaped by local understandings of morality, historical legacies and individual experiences [41]. In Sierra Leone, conversations around fairness often revolve around the widespread assumption that access to resources relies on having a strong *sababu*, that is, a connection with people in positions of power. Lacking a *sababu* is frequently cited as a key reason why large numbers of Sierra Leoneans live in poverty, with limited access to jobs and basic healthcare [42]. Strong critiques of “connectocracy”, furthermore, are paralleled by assumptions that nepotism is the only way to access resources. Beliefs about fairness were also central to the unfolding of the ten year civil war, for example as discontent surrounding chiefly abuse of powers in terms of forced community labour demanded of young men, featured prominently in combatants’ accounts of their motivations for joining rebelling forces [43].

In clinical research the notion of fairness emerges at several stages of the research, from concerns about equal treatment of control and treatment groups, to issues of compensation and the addressing of potential medical complications. In the initial stages of trial design, which this paper focuses on, engaging with local ideas of fairness was an especially important in trialists’ discussions surrounding the design of a volunteer recruitment strategy. The senior investigators and trial team expected the issue of participant recruitment in EBOVAC Salone to be challenging, especially in an epidemic context, in a country with little experience of clinical trials. Consequently, the design of a recruitment strategy was discussed at length, combining senior trialists’ expertise of vaccine trials in other contexts with conversations with local staff and anthropological insights. As Ezekiel et al. have pointed out: fair selection of the study population is a key benchmark of ethical clinical research in developing countries [44]. As such, the trial team had to consider not only their own understandings of what would be an effective trial design that would ensure that the results it provided would be scientifically valid and representative of the community as a whole, but also how the Kambia community would perceive the selection of volunteers.

Initial discussions centred on a number of design options, including individuals coming directly to the clinic to volunteer or having a key authority figure canvass for volunteers. Given the context discussed above, these options raised concerns that the trial would be liable to complaints of unfairness, as well as the fact that the enrolled participants would be likely to be unrepresentative of the population as a whole. Firstly, given assumptions that access to resources is assumed to be based on “connectocracy”, there was the potential that, given the limited number of participants required, people could have assumed that the “big ones” were picking themselves and those they knew. On the other hand, using key authority figures to compile a list of volunteers, or asking key stakeholders to be the first volunteers could have led to accusations of forced participation. Given Sierra Leone’s history of conflict, the potential for participant recruitment to be likened to forced conscription also added sensitivities for the trial recruitment process.

In consideration of these concerns, and after extensive consultation with the principal investigators, trial sponsor and the trial manager, the community liaison team opted for a public lottery of household numbers followed by individual visits through which people would be offered the opportunity to volunteer in the vaccine trial. This involved inviting community members to observe and take part in the selection of 100 households (to begin with) from a bag containing all household numbers in Kambia town.^6^ These households were then visited individually by community liaison officers who explained in detail the purpose and procedures of the trial and voluntary nature of the process and provided these randomly selected households with the opportunity to volunteer.

Notions of fairness, like power, are contested and there are limitations in the extent to which any design can avoid criticism. For example, as initial fears surrounding the new vaccine trial subsided, some community members expressed worries that the lottery system would not be able to taking into account those who were eager to take the vaccine.^7^ However, as one civil society activist noted, using a lottery system meant that “*bad name done komot dae*” (the trial has avoided a bad reputation).^8^ Had it not been done through a public lottery, she argued, allegations of unfairness would have been widespread.^9^

### Trust

A major challenge in preparing for this vaccine trial in Sierra Leone during the Ebola epidemic was the high levels of fear and mistrust which, as discussed, had develop through a history of oppressive rule and conflict. The epidemic was accompanied by a plethora of stories and rumours that starkly exposed the lack of confidence in government authorities, medical practitioners, and external agencies [6, 45, 46]. This was made clear by the widespread belief that Ebola was a man-made, population control strategy in view of the next Presidential elections; that people were killed inside ambulances by being asphyxiated by chlorine; or that new cases were fabricated in order for Ebola response workers to profit from the protracted crisis [6, 47] These rumours have too often been treated as a sign that populations are “misinformed” at best, “ignorant” at worst, and are thus simply in need of better information in order to change their behaviour [18]. However, these rumours reflect broader anxieties rooted in a much deeper socio-political context, and explain resistance to the Ebola response at the height of the epidemic. Anxieties surrounding the government’s and international partners’ plans reveal fractures in citizens’ trust in the healthcare sector, which is seen as corrupt and inefficient, and reflects the fragility of the social contract rebuilt in the aftermath of Sierra Leone’s civil war. Understanding public perceptions, rumours and concerns in this fragile context and creating multiple forums for dialogue and trust-building, were an essential foundation for more trial-specific community engagement.

As communities learned about the planned vaccine trial in Kambia, the ethnography revealed that concerns surrounding the Ebola outbreak and the response to it were initially transposed to ideas about what the trial would involve. One evening, when the social science team discussed perceptions of the vaccine trial with a group of young men, one of them said he had a concern he had previously been ashamed to share. He said that he had heard that there was a “world blood bank” that was lacking type O blood. He argued that survival at the Ebola treatment centres had been determined by one’s blood type, as those with type O blood were killed for their blood. Survivors were those whose blood type was not needed by the world blood bank and who were thus allowed to return to their communities alive. He reported that he had been afraid during the Ebola outbreak because he knew his blood type was O. Having heard in several community meetings that blood would be taken during the process of the vaccine trial, he asked whether this was actually a continuation of the blood stealing that he believed had been going on since the outbreak of Ebola.

In other ethnographic encounters in Kambia’s key congregation areas, the social science team frequently recorded links being drawn between the epidemic and the vaccine study by referring to the impending trial as “Ebola Phase Two”. As with the young man’s concern about blood stealing, this title referred to the concern that the trial might be a plan to “finish off” those who had managed to escape death during the outbreak. As one trial participant put it when discussing the rumours he had heard that “[the vaccine] is a slow poison the white man has made to kill us”.^10^

While maintaining a commitment to the confidential nature of research conversations, the social science team alerted the community liaison team when these fears and articulations of mistrust emerged. The community liaison team responded by visiting areas such as the town’s market where the idea of the vaccine being “Ebola Phase Two” had taken root. Being aware of the particular nature of anxieties surrounding blood-donation in the context of the Ebola outbreak meant that the community liaison team encouraged debates and conversations through community meetings, one-on-one conversations and radio shows to discuss the role of blood taking in the vaccine trial. They provided explicit information about the destination and use of blood samples and the fact that samples could be destroyed if the participant so wished once the study was over. They also invited questions, challenges and suggestions. Representatives of different societal groups who had previously attended community engagement events hosted their own meetings, which the community liaison team attended as guests, encouraged open and often heated debates, and also enabled the creation of spaces for expressing and confronting anxieties rather than simply rejecting them as misinformation.

In another example, community meetings and ethnographic encounters brought up the issue of the insurance that would be provided for participants in case of any long-term side-effects from the vaccine. This had sparked worried conversations about the likelihood that if the trial provided insurance it meant the trialists expected people to die in the process. Concerns identified by the social science team were fed back to the community liaison team. The team developed a strategy to help allay fears around this rumour, including a new message about the provision of insurance that also emphasised the safety of the vaccine. An influential individual in that particular community, who had worked with the community liaison team, volunteered to assist with disseminating the message and dispelling the rumour through discussions in the area where the rumour had emerged.

Such rumours represent more generalised concerns about medical interventions; they are not simple misunderstandings but are rather rooted in histories of exploitation and mistrust. Therefore, whilst immediate messaging was seen to be important, the main role of the teams was to develop communication to understand community concerns, and the drivers behind these concerns, and to work to build trust through ongoing active and inclusive dialogue.

## Discussion

Carrying out vaccine trials in the context of an infectious disease epidemic with high mortality in a developing county recovering from years of internal conflict brings significant challenges. The EBOVAC Salone trial was set up during the Ebola outbreak as affected populations were trying to make sense of the disease and its devastating impact on families and communities. In addition, the epidemic has laid bare, and sometimes exacerbated, mistrust in the healthcare system, elected officials and external health interventions. In order to understand some of the concerns surrounding the trial, it was crucial that the teams took into account how the Ebola epidemic has affected social relations and power as well as local ideas about the Ebola response, including interactions with Western medicine and experiences of community engagement. Even more broadly, it was important for the two teams to work together to engage effectively with the community dynamics and power constellations highlighted by anthropological research. Such understandings of the community in which the trial was being set up have helped the teams to ensure that communities and participants are given an opportunity to voice concerns and to work with them to address mistrust. Knowledge of contested power, for example, allowed the team to listen to the voice of “small leaders” and to take into account their potential to disengage from the trial.

This paper has offered illustrations of how social science and community liaison team worked in concert to shape engagement strategies and volunteer recruitment mechanisms. The examples discussed here show the value of research-driven communications and offer important lessons for future trials. Firstly, they demonstrate the importance of real-time social science research in the setting up of a vaccine trial. Social science researchers can act as independent “eyes and ears of the trial”, listening to fears, concerns and suggestions. Secondly, an in depth understanding of community power dynamics highlights the importance of diversifying communication methods and avenues for engagement. As shown by the concerns of the “small” community leaders, contestations of power can be muted and hidden but can nonetheless affect the relationship between the vaccine trial and the community. As such, using a variety of communication channels, rather than relying solely on established leadership, can ensure messages reach different sectors of the community. Thirdly, an understanding of community dynamics and local social norms through ongoing dialogue can help inform the set-up of a trial from the start as evidenced by the establishment of a public lottery for volunteer recruitment. Finally, a focus on listening to and understanding rumours revealed deeper concerns about health interventions stemming from histories of mistrust, rather than simply being “misunderstandings”.

## Conclusion

This paper reveals that lessons learned from ethnographies of other trials about political and social inequities, social relations and rumours and concerns have been heeded. However, they also highlight very specific and local concerns in the context of the Ebola epidemic, which can only be revealed by on-going and “close to the ground” social science research and effective and dynamic community engagement.

## Notes

More information on the EBOVAC Trial can be found at: http://www.ebovac.org and https://clinicaltrials.gov/ct2/show/NCT02509494?term=EBOVAC&rank=1.

## Abbreviations

COMAHS: College of Medicine and Allied Health Sciences
LSHTM: London School of Hygiene and Tropical Medicine

## Footnotes

1 The vaccine being used as the active control is the WHO-prequalified Meningitis ACWY vaccine. This is given as a prime dose at Day 1 and a placebo as the boost dose, except in children under the age of 2 years who are given 2 doses of the active control vaccine.

2 Chinese gunpowder tea

3 Palm wine

4 Commercial motorbikes

5 These interpretations, and the expectations that they raised, must also be understood in the context of the traditional practice of paying ‘kola’, a ‘greeting gift’, to authorities and gatekeepers, such as chiefs in order to begin community consultations, as well as the widespread payment of traditional authorities and land custodians in order to facilitate business, such as in the mining industry [48, 49].

6 Before carrying out a lottery, the social science team mapped Kambia Town using Global Positioning System (GPS) technology and produced a list of all households in the town, as this was not available at the time.

7 Community Focus Group Discussion, Kambia, 23 November 2015

8 Key Informant Interview, Kambia, 19 October 2015

9 The fact that there may have been accusations of unfairness seems to suggest a particular eagerness (or the expectation of eagerness) to participate amongst Kambia residents in the initial stages, and during the epidemic. This begs the question of what people’s motivation for joining may have been, what perceived incentives may play a role and whether certain selection biases may have been at play. Determining what factors influence participants’ decisions to enroll in the EBOVAC trial is a key component of the social science research taking place throughout the running of the trial. This reflects the contribution that qualitative research can make by highlighting potential ethical issues as they arise during a clinical research project.

10 Exit Interview, Kambia, 20.10.2015
